# African Tick-Bite Fever in Traveler Returning to Slovenia from Uganda

**DOI:** 10.3201/eid2210.160650

**Published:** 2016-10

**Authors:** Petra Bogovic, Stanka Lotric-Furlan, Misa Korva, Tatjana Avsic-Zupanc

**Affiliations:** University Medical Center Ljubljana, Ljubljana, Slovenia (P. Bogovic, S. Lotric-Furlan);; Institute of Microbiology and Immunology, Ljubljana (M. Korva, T. Avsic-Zupanc)

**Keywords:** African tick-bite fever, travelers, Slovenia, Uganda, ticks, vector-borne infections, rickettsia

**To the Editor:** African tick-bite fever (ATBF) is a well known disease in travelers to sub-Saharan Africa (*1*). The causative agent, *Rickettsia africae*, is transmitted to humans by ticks of the genus *Amblyomma* (*1*,*2*). *R. africae* has been isolated or detected in ticks, humans, or both in 22 sub-Saharan countries (*3*). Most ATBF cases have been described in tourists returning from countries to which it is endemic, most often from South Africa, Zimbabwe, and Botswana (*4*). We report a case of ATBF in a Slovenian traveler returning from Uganda.

In June 2015, a 29-year-old Slovenian man without underlying illnesses sought care at the Department of Infectious Diseases, University Medical Centre Ljubljana (Ljubljana, Slovenia). He had a 1-day history of fever up to 38°C without chills, 5 days after returning from a 2-week trip to Uganda. He had received vaccines against yellow fever and viral hepatitis A before traveling and did not use antimalarial prophylaxis during his stay in southwestern Uganda. A day before he left Uganda, he noticed a tick bite on his left upper abdomen.

At initial examination, he appeared well. He had a temperature of 37.8°C, pulse rate 75 beats/min, and blood pressure 120/80 mm Hg. Skin examination was remarkable for a solitary papular lesion at the site of tick bite surrounded by a small erythematous halo associated with discrete lymphangitic streaking and painful enlarged left axillar lymph nodes. Results of initially performed routine laboratory tests were normal.

On day 5 of illness, the man was still febrile, with a temperature up to 39°C. Papular skin lesion had developed a dark brown crusted center (compatible with a tache noire), and some new discrete asymptomatic pale papular skin lesions appeared on his left leg and arm. Repeat laboratory testing indicated only mildly increased serum C-reactive protein (16.0 mg/L [reference <5 mg/L]).

The clinical course improved rapidly after treatment began with doxycycline. Fever resolved in 2 days, and enlarged lymph nodes and skin lesions resolved completely within 14 days.

Microbiological procedures to detect for infection with tick-transmitted pathogens were performed to elucidate the cause of the illness. The PCR for amplification of a 470-bp fragment of citrate synthase gene was performed according to a previously published protocol (*5*). DNA was extracted with QIAamp DNA Mini Kit (QIAGEN, Hilden, Germany) from whole blood and the crust of the eschar collected on day 5 of illness. In addition, serum samples were tested by indirect immunofluorescent assay for specific IgG and IgM against *Francisella tularensis* and *Rickettsia* spp. (spotted fever and typhus group) 5 days and 10 weeks after onset of fever.

Diagnosis of ATBF was affirmed by positive PCR result from the crust of the eschar; further sequence analysis revealed the infection with *R. africae*. Serologic testing demonstrated seroconversion of IgG to *R. conorii* and *R. rickettsii*, which cross-reacts with *R. africae* (negative immunofluorescent assay IgG titer at initial testing and 1:1,024 for *R. conorii* and *R. rickettsii* 10 weeks later) (*6*). Thick and thin blood smears were negative for malaria.

ATBF is the second most well-established cause of febrile illness among travelers to sub-Saharan Africa, after malaria. Usually it manifests by fever (59%–100% of cases), headache (62%–83%), eschar (53%–100%), lymphadenopathy (43%–100%), and rash (15%–46%). The clinical and laboratory findings in the patient reported here were similar to those previously reported among *R. africae*–infected patients (*1*). In the acute phase of illness, a biopsy and culture from an eschar, as well as PCR, are the most suitable methods to confirm the ATBF diagnosis. In this case, ATBF was proven by PCR and subsequent sequencing from a crust sample but not from whole blood and seroconversion of IgG.

The first information about *R. africae* in ticks in Uganda was published in 2013 by Lorusso et al. (*7*), but previously *R. conorii* also was found (*8*). The prevalence rate of *R. africae* infection among *Amblyomma variegatum* ticks in Uganda was 97.1% (*9*). Recently, Proboste et al. established the presence of previously undetected tickborne pathogens in rural dogs and associated ticks in Uganda. Tick species *Haemaphysalis leachi*, *Rhipicephalus* spp., and *A. variegatum* were infected by *Rickettsia* spp. (18.9%), including *R. conorii* and *R. massiliae*; by *Ehrlichia* spp. (18.9%), including *E. chaffeensis*; and by *Anaplasma platys* (*10*).

Our MEDLINE literature search found no previous descriptions of human *R. africae* infection in Uganda. This case indicates that ATBF should be included as a possible diagnosis in persons with febrile illness who have traveled to Uganda, a well-known tourist destination.
